# How Digital Diaries Are Changing Communication and Recovery in the Intensive Care Unit and Beyond

**DOI:** 10.2196/92661

**Published:** 2026-02-20

**Authors:** Jenna Congdon

**Keywords:** intensive care units, patient diaries, post–intensive care syndrome, mental health, survivors, professional-family relations, communication, trauma-informed care, critical care, patient-centered care, digital health, rehabilitation, recovery of function

**Key Takeaways**

- Digital intensive care unit (ICU) diaries help patients and families reconstruct “lost time,” supporting psychological recovery after critical illness.
- By centralizing updates and humanizing care, digital diaries can improve communication between ICU staff and families while reducing interruptions to clinical work.
- Digital diaries can be a low-burden, high-impact tool supporting human-centered, trauma-informed ICU care long after discharge.

When patients finally leave after a long stay in the intensive care unit (ICU), they may feel as if they’ve lost weeks of their life. Perhaps they were unconscious, confused, or otherwise unable to process the whirlwind of illness and medical interventions that occurred during their stay. Additionally, clear and consistent communication between patients’ family members and medical staff supports improved patient outcomes, but remains a perennial challenge in the ICU due to the complex nature of patients’ medical courses.

A digital diary—a secure app-based journal that allows staff and families to log daily events with messages, photos, and videos to chronicle a patient’s hospital stay—addresses both these concerns. As they recover, patients can reference the diary to process the complex experiences they may have had in the ICU. Digital diaries can improve mental and physical health outcomes for patients, foster stronger communication between staff and family members, and serve as a powerful tool to support patients’ recovery and a poignant reminder of the love and support they received along the way.

## Digital Diaries: A Valuable Record of Time Lost

Jurriaan van Rijswijk, MSc, founder of Games for Health [1], a company that develops app-based games to improve health outcomes, saw an opportunity to both improve staff-family communication and support patients’ mental health after an ICU stay. Together with medical staff and researchers, he developed the Post-ICU digital diary app [2].

The app was initially launched at Erasmus University Medical Center in Rotterdam, Netherlands, to bridge the gap between patients and distant family members during the COVID-19 pandemic. It has since expanded; at the time of this writing, the diaries are in use across 24 hospitals, primarily in Europe.

## Post–Intensive Care Syndrome

Patients often experience a constellation of mental health challenges after an ICU stay, particularly if their hospitalization was long or complicated [3]. In one study, 46% of patients experienced anxiety symptoms following their hospital stay. In addition, 40% reported feeling depressed, 22% were diagnosed with posttraumatic stress disorder (PTSD), and 18% of patients faced all three. Morbidity was 47% higher for patients experiencing depression compared to patients without depression in the two years following discharge from the ICU [4].

This decline in mental health, often accompanied by negative physical and cognitive changes, is referred to as post–intensive care syndrome (PICS) [4]. This syndrome not only makes it difficult to return to daily activities, but can threaten long-term survival and quality of life [5]. Ongoing support and intervention after discharge is needed to treat these issues and ensure patients’ maximal recovery.

After digital diary implementation, patients had fewer instances of post-ICU anxiety and depression, and their loved ones experienced lower rates of PTSD after their hospital stay [6]. For some ICU survivors, rereading their digital diary helped them cope with the aftermath of a traumatic illness [7].

## Improving Staff-Family Communication

During an ICU stay, all focus goes to the patient, and communication between staff and family members can be complex—on one side, the worried loved ones; on the other, busy nurses and doctors. When details get missed or misunderstood, both sides end up frustrated, and patient outcomes may suffer. When communication between both parties is centralized, families are more engaged in their loved ones’ care and medical errors may be reduced [8].

The diaries streamline communication between staff and family members, saving time for everyone and ensuring that family members feel involved even when they can’t be physically present. The app is easily activated by a nurse through the patient’s medical record. Nursing staff write in the diary every morning, helping to humanize care with heartfelt, nonclinical notes alongside medical details. Family members know they can simply check the app for updates on their loved one rather than call the hospital, leading to fewer patient care interruptions.

## Notes on Adoption and Challenges

Feedback from staff and families has been largely positive.

“Families love the solution,” Jurriaan says, and he notes that “the app has a feedback button...they mostly use it to compliment us.”

Concerns from family members largely center around two topics: feeling unsure of what to write and struggling to print the diary as a keepsake or convert it to a PDF to share. The former has been addressed with simple in-app writing prompts, the latter with straightforward tech support.

“It is a super simple app,” Jurriaan shares. “It has three buttons, one to write and to add pictures, one to read, and one button for information.”

This decreases barriers for users with low levels of digital literacy [7]. Furthermore, the diary remains accessible after discharge, making it easy to share with therapists or other caregivers.

Hospital administrators sometimes report qualms regarding data privacy. With the Post-ICU app, the family maintains control of who can access the diary, and careful security measures are in place to protect patient data. Jurriaan points out that compared to a physical journal, “...it’s much safer. With a physical journal, you can’t control who touches it, who reads it. It is a hygiene problem and a security problem. A digital journal is safer.”

And while nurses have expressed concern about lacking the time to write entries, they nonetheless report that the diaries are a worthwhile addition to patient care, fostering a sense of human connection [9].

To address potential burden, the implementation process should be participatory and include end user medical staff to ensure ongoing relevance as workflows adapt [10]. Responsibility for diary entries can be shared across medical disciplines to increase overall participation.

For some patients, digital diaries can provoke sadness and loss; in one study, roughly one-third of patients surveyed reported rereading their ICU diary as a particularly distressing and painful experience [11]. Staff and families should use careful judgment around how and when to share the diaries with recovering patients.

## Digital Diaries Support Human-Centered ICU Care

Jurriaan shares the story of an on-call nurse he worked with, who attended a beloved patient’s funeral:

“...to her surprise, some of the [recordings] she made in the diary for that person were being read in front of everyone. How beautiful...it was shared with the entire audience during the funeral. I believe that’s incredible, that although the patient died, it is still of use...for the family.”

Digital diaries are more than clinical documentation. They are trauma-informed care tools that can serve as powerful sources of ongoing support and inspiration for families, medical providers, and patients long after discharge. They can help patients feel seen as people rather than clinical cases, validate feelings at a time when emotions are high, and provide staff with a respectful way to connect with their patients on a human level.

Recovery goes far beyond just physical survival. Regaining lost memories and processing traumatic events can allow patients and their loved ones to face life after the ICU with resilience. For patients who lost weeks of their lives, a digital diary doesn’t just fill in the gaps; it gives the story back to them. Thoughtfully implemented, digital diaries represent an elegant intervention supporting total-person healing during the ICU stay and beyond.

## References

[1] Games for health.

[2] Post-ICU.

[3] Chang CS, Tsai FJ, Liao CH (2025). Associations between elevated rates of depression, anxiety, and PTSD among ICU survivors and increased mortality and readmissions. Brain Behav.

[4] Hatch R, Young D, Barber V, Griffiths J, Harrison DA, Watkinson P (2018). Anxiety, depression and post traumatic stress disorder after critical illness: a UK-wide prospective cohort study. Crit Care.

[5] Voiriot G, Oualha M, Pierre A (2022). Chronic critical illness and post-intensive care syndrome: from pathophysiology to clinical challenges. Ann Intensive Care.

[6] McIlroy PA, King RS, Garrouste-Orgeas M, Tabah A, Ramanan M (2019). The effect of ICU diaries on psychological outcomes and quality of life of survivors of critical illness and their relatives: a systematic review and meta-analysis. Crit Care Med.

[7] van Mol MMC, Tummers N, Leerentveld C, Tieben R, Buise M (2024). The usability of a digital diary from the perspectives of intensive care patients’ relatives: A pilot study. Nurs Crit Care.

[8] Khan A, Spector ND, Baird JD (2018). Patient safety after implementation of a coproduced family centered communication programme: multicenter before and after intervention study. BMJ.

[9] Haakma T, Tieben R, Sleven B, Buise M, van Mol M (2022). Experiences of nurses with an innovative digital diary intervention in the intensive care unit: a qualitative exploration. Intensive Crit Care Nurs.

[10] Schol CMA, van Mol MMC, Ista E (2025). Developing implementation strategies for digital ICU diaries targeting ICU professionals: an implementation mapping approach. Implement Sci Commun.

[11] Flahault C, Trosdorf M, Sonrier M (2021). ICU survivors experience of ICU diaries: an ancillary qualitative analysis of the ICU diary study. Crit Care Explor.

